# *Aspergillus fumigatus* Extracellular Vesicles Display Increased *Galleria mellonella* Survival but Partial Pro-Inflammatory Response by Macrophages

**DOI:** 10.3390/jof9050541

**Published:** 2023-05-04

**Authors:** Mateus Silveira Freitas, Tamires Aparecida Bitencourt, Caroline Patini Rezende, Nubia Sabrina Martins, Thales de Mileto Henrique Dourado, Carlos R. Tirapelli, Fausto Almeida

**Affiliations:** 1 Department of Biochemistry and Immunology, Ribeirão Preto Medical School, University of São Paulo, Ribeirão Preto 14049-900, SP, Brazil; 2 Department of Pharmacology, Ribeirão Preto Medical School, University of São Paulo, Ribeirão Preto 14049-900, SP, Brazil; 3 Laboratory of Pharmacology, Department of Psychiatric Nursing and Human Sciences, College of Nursing of Ribeirão Preto, University of São Paulo, Ribeirão Preto 14040-902, SP, Brazil

**Keywords:** *Aspergillus fumigatus*, extracellular vesicles, inflammation, innate leukocytes, protection

## Abstract

Fungal extracellular vesicles (EVs) mediate intra- and interspecies communication and are critical in host–fungus interaction, modulating inflammation and immune responses. In this study, we evaluated the *in vitro* pro- and anti-inflammatory properties of *Aspergillus fumigatus* EVs over innate leukocytes. *A. fumigatus* EVs induced a partial proinflammatory response by macrophages, characterized by increased tumor necrosis factor-alpha production, and increased gene expression of induced nitric oxide synthase and adhesion molecules. EVs induce neither NETosis in human neutrophils nor cytokine secretion by peripheral mononuclear cells. However, prior inoculation of *A. fumigatus* EVs in *Galleria mellonella* larvae resulted in increased survival after the fungal challenge. Taken together, these findings show that *A. fumigatus* EVs play a role in protection against fungal infection, although they induce a partial pro-inflammatory response.

## 1. Introduction

*Aspergillus fumigatus* is the main causative agent of aspergillosis, which can be a fatal disease [1]. *Aspergillus* are saprophytic organisms that release many airborne conidia [2,3]. The first line of defense against inhaled *A. fumigatus* conidia are resident macrophages, which interact with pathogen-associated molecular patterns from swollen conidia and promote cytokine release, phagocytosis, and intracellular killing [4]. Soluble factors, such as CXCL1 and CXCL2, released by resident macrophages, and other immune cells, recruit neutrophils to the site of infection [5]. Neutrophils are pivotal in conidia elimination and host protection [6].

Extracellular vesicles (EVs) are released by virtually all living organisms. The spherical structures formed of a lipid bilayer membrane transport several biomolecules [7,8]. Several species of fungi produce EVs [9,10,11,12,13,14] that have important biological functions [15]. Although their role in the infective process is still not clear, fungal EVs have been reported to have an effect on host immune responses and could also mediate intraspecies fungal communication [16,17]. EVs from *A. flavus*, *Paracoccidioides brasiliensis*, and *Trichophyton interdigitale* have strong proinflammatory activity for macrophages, with the increased release of proinflammatory mediators such as nitric oxide (NO), IL-12p40, IL-12p70, IL-6, TNF-α, IL-1α, and IL-1β, augmented phagocytosis and fungal killing, and activation toward inflammatory or M1 macrophages [13,18,19]. On the other hand, EVs from *Histoplasma capsulatum* display anti-inflammatory activity for macrophages and might reduce phagocytosis, intracellular killing, and the production of reactive oxygen species (ROS) [20]. These studies have demonstrated that fungal EVs have different actions on innate leukocytes, priming these cells to better control fungal infection or foster fungal growth.

Since EVs play an up- or down-modulatory role, in this study, we investigated the role of *A. fumigatus* EVs in the activation status of macrophages, neutrophils, and monocytes. These cells are pivotal in immune responses against *A. fumigatus*. Our data suggest that *A. fumigatus* EVs play a role in protection against fungal infection, inducing a partial pro-inflammatory response. Thus, our results suggest that *A. fumigatus* EVs could be exploited in antifungal therapies.

## 2. Materials and Methods

### 2.1. Ethics Statement

The protocols involving human cells used in this study were approved by the Research Ethics Committee of the University Hospital, Ribeirao Preto Medical School at the University of Sao Paulo (protocol 4.277.966), and informed written consent from all participants was obtained.

### 2.2. A. fumigatus Strain and Growth Conditions

*A. fumigatus* strain CEA17 was cultivated as described previously [16], with slight modifications. Freshly thawed vials containing conidia were seeded in a complete agar malt medium for 7 days at 37 °C. These plates were washed with PBS followed by centrifugation and filtration through sterile Miracloth (Millipore, Billerica, MA, USA) to obtain conidia suspensions. Conidia concentration was determined using a Neubauer chamber. Approximately 1 × 10^7^ conidia were seeded in agar minimal medium [19]. After 2 days of incubation in 37 °C, the plates were washed with sterile PBS. EVs were then isolated and purified.

### 2.3. Isolation and Purification of EVs

Plates containing 2-day-old *A. fumigatus* cultures were washed twice with 5 mL of sterile PBS with the aid of inoculation loops. The recovered solution was filtered through sterile Miracloth (Millipore) and centrifuged at 5000× *g* for 15 min at 4 °C. The supernatant was recovered and centrifuged again at 15,000× *g* for 15 min at 4 °C. Supernatant concentration was performed in an Amicon system. Another centrifugation at 15,000× *g* for 15 min at 4 °C was performed, and the supernatant was filtered through a 0.45 µm filter. An ultracentrifugation was performed at 60,000 rpm for 1 h at 4 °C in a TLA-120.2 rotor (Beckman Coulter). The pellet was resuspended in sterile PBS. The due rotor fixed angle of rotation was 30 degrees; 60,000 rpm are equivalent of 98,800× *g* in its minimum radius. The obtained EVs were subjected to nanoparticle tracking analysis using a NanoSight NS300 system (Malvern Instruments, Malvern, UK) to verify their size, distribution, and concentration.

### 2.4. Culture of Macrophages

AMJ2-C11 and RAW 264.7 macrophages were maintained in Dulbecco’s modified Eagle’s medium (Sigma–Aldrich, St. Louis, MO, USA) supplemented with 10% fetal bovine serum (HyClone, Logan, UT, USA) and 1% penicillin-streptomycin (Gibco, Thermo Fisher Scientific Inc., Waltham, MA, USA) at 37 °C in a 5% CO_2_ atmosphere. Cells were cultivated until confluence and detached from the flask bottom with Trypsin-EDTA solution (Sigma–Aldrich). The cells were counted in a Neubauer chamber and plated in 96- or 48-well culture plates (depending on the assay performed) and in a fetal bovine serum-free medium.

### 2.5. Isolation of PBMCs

Blood withdrawn under authorized supervision was collected using Vacutainer heparin collection tubes. PBMC separation was performed using Histopaque-1077 (Sigma–Aldrich). Briefly, collection tubes were centrifuged at 500× *g* for 20 min at 20 °C. The separated plasma was discarded. The remaining samples were diluted in PBS up to 15 mL. The solution was carefully layered over 6 mL of Histopaque-1077 solution and centrifuged at 500× *g* for 30 min at 20 °C. The rings formed with PBMCs were harvested and washed with PBS and centrifuged at 500× *g* for 10 min at 20 °C. The cells were resuspended in RPMI 1640 medium (Sigma–Aldrich) with 1% penicillin-streptomycin (Gibco, Thermo Fisher Scientific Inc.) lacking fetal bovine serum.

### 2.6. Cytokine Measurement

AMJ2-C11 or RAW 264.7 macrophages were plated in 96-well plates (3 × 10^4^ cells/well) at 37 °C in a 5% CO_2_ atmosphere and rested overnight. Cells were stimulated with EVs (10^7^ to 10^10^ EVs/mL), LPS (1 µg/mL) or medium alone for 24 h at 37 °C in a 5% CO_2_ atmosphere. After incubation, the supernatant was tested for the presence of TNF-α, IL-6, and IL-1β via enzyme-linked immunosorbent assay using an OptEIA kit (Pharmingen, San Diego, CA, USA), according to the manufacturer’s instructions. For the PBMCs, the cells were plated in 96-well plates (2 × 10^5^ cells/well) at 37 °C in a 5% CO_2_ atmosphere, and soon after they were stimulated with EVs (10^9^ to 10^10^ EVs/mL), LPS (1 µg/mL) or medium alone for 24 h at 37 °C in a 5% CO_2_ atmosphere. The presence of human cytokines (TNF-α, IL-6 and IL-10) was assessed using an OptEIA kit as described above. Absorbance was read at 450 nm in the Power Wave-X microplate scanning spectrophotometer (BioTek Instruments, Inc., Winooski, VT, USA).

### 2.7. Nitric Oxide (NO) Measurement

NO was measured in the supernatant of AMJ2-C11 cells plated (3 × 10^4^ cells/well) in 96-well plates at 37 °C in a 5% CO_2_ atmosphere stimulated for 24 h with EVs (10^9^ and 10^10^ EVs/mL), Pam3CSK4 (1 µg/mL), or medium alone. NO was quantified using the Griess method [21]. Briefly, 50 µL of cell supernatant was mixed with the same volume of Griess reagent (1.0% sulfanilamide, 0.1% naphthalenediamine dihydrochloride, and 2.5% H_3_PO_4_) at room temperature for 10 min in 96-well plates. Absorbance at 550 nm was read using a Power Wave-X microplate reader (BioTek Instruments, Inc.). The absorbance was converted to micromolar (µM) NO based on a standard curve generated using known concentrations of NaNO_2_.

### 2.8. Detection of O_2_^•−^ by Lucigenin Enhanced Chemiluminescence

Superoxide levels were measured in AMJ2-C11 and RAW 264.7 culture supernatants using the lucigenin-derived chemiluminescence assay. Both cells were plated (3 × 10^4^ cells/well) in 96-well plates at 37 °C in a 5% CO_2_ atmosphere and stimulated for 24 h with EVs (10^9^ and 10^10^ EVs/mL), LPS (1 µg/mL), or medium alone. The reaction was carried out by the addition of 50 µL of the sample in the assay buffer, containing 50 mM KH_2_PO_4_, 1 mM EGTA, 150 mM sucrose, (pH 7.4) 5 µM lucigenin in 250 µL total volume. The addition of 0.1 mM of NAD(P)H started the reaction. Luminescence was measured with an Orion II luminometer (Berthold Technologies, Pforzheim, Germany). Blank was subtracted from each sample reading. The production of superoxide was expressed as relative light unit (RLU)/3 × 10^4^ cells.

### 2.9. qPCR

AMJ2-C11 and RAW 264.7 macrophages were plated in 48-well plates (4 × 10^5^ cells/well) at 37 °C in a 5% CO_2_ atmosphere and rested overnight. The cells were stimulated with EVs (1 × 10^10^ EVs/mL), LPS (1 µg/mL), LPS + Pam3CSK4 (500 ng/mL each), or medium alone for 6 and 24 h at 37 °C in a 5% CO_2_ atmosphere. The supernatant was discarded, and the cells were used for RNA extraction using the Illustra RNAspin Mini RNA isolation kit (GE Healthcare, Chicago, IL, USA) following the manufacturer’s instructions. RNA concentration and quality were measured using a nanophotometer (Implen, Westlake Village, CA, USA). Before complementary DNA (cDNA) synthesis, RNA was treated with DNase (Sigma–Aldrich). cDNA synthesis was performed using a high-capacity cDNA reverse-transcription kit (Applied Biosystems, Foster City, CA, USA), following the manufacturer’s instructions. Quantitative real-time PCR was performed using SYBR Green (Applied Biosystems) in the Step One Plus platform. The relative expression of transcripts was quantified using the ΔΔCt method and normalized relative to Gapdh expression. The primers used are listed in Table 1.

### 2.10. Macrophage Adhesion Assay

High binding plates were coated with fibrinogen (20 µg/mL; Sigma–Aldrich) overnight at 4 °C. The plate was washed with sterile PBS, and RAW 264.7 macrophages were seeded at 6 × 10^4^ cells/well. Immediately after seeding, the cells were treated with LPS (1 µg/mL) or EVs (1 × 10^10^ EVs/mL). The plate was kept in a humidified incubator at 37 °C in a 5% CO_2_ atmosphere for 24 h. Each well was washed three times with 200 µL of PBS to remove non-adherent cells. After the last wash, the cells were incubated with a solution of 100 µM Resazurin (Sigma–Aldrich) in 150 µL of incomplete DMEM. After 4 and 24 h of incubation at 37 °C in a 5% CO_2_ atmosphere, the plate was read in a plate reader (BioTek Instruments, Inc.) at 570 and 600 nm. The percentage of the reduced form of Resazurin was calculated following the manufacturer’s instructions. Increased levels of reduced Resazurin were an indication of more cells in each well.

### 2.11. Isolation and Culture of Neutrophils

Healthy volunteers provided consent to serve as blood donors. Blood was withdrawn under authorized supervision. The blood was collected in tubes containing EDTA, and neutrophils were isolated by a Percoll gradient. Briefly, the gradient was layered by adding Percoll concentrations of 72, 64, 54, and 45%. Blood sample was added above the 45% layer. The gradient was centrifuged at 650× *g* for 32 min. The layer of polymorphonuclear cells was collected, centrifuged at 400× *g* for 10 min, and plated (5 × 10^5^ cells/well). The cells were stimulated with EVs (10^9^ or 10^10^ EVs/mL) overnight. As the positive control, cells were stimulated with 10 µM phorbol myristate acetate (PMA) for 2 to 4 h. PMA is a known inducer of NETosis [25].

### 2.12. Detection of NETs by Flow Cytometry

Cultured neutrophils were labeled with FVS780 viability stain (BD Biosciences, San Diego, CA, USA) and Sytox Green (Thermo Fisher Scientific). Fluorescence was detected using a FACS Melody device (BD Biosciences, San Jose, CA, USA). Analysis was performed using the FlowJo software (Becton Dickinson and Company, Franklin Lakes, NJ, USA).

### 2.13. G. mellonella Survival Assay

*G. mellonella* larvae were kept in sterile glass flasks with modified lids, with a hole in the center covered with an ultra-fine stainless steel wire mesh for better ventilation. They were maintained in a BOD SL-200 incubator (Solab cientifica) in the dark with a controlled temperature of 28 °C. They were fed with an in-house-produced mixture of bee wax, beer yeast, soy, honey, powder milk, and corn meal. *G. mellonella* survival was assayed as previously described [19]. Ten healthy *G. mellonella* larvae of similar weight (approximately 240 to 300 mg) were used in each group. Each larva was inoculated with 57 µL of 10^10^, 10^9^, or 10^8^ EVs, or with PBS as the control, into the last left proleg, directly on the hemocoel. After 48 h, all larvae were infected with 10 µL of a solution containing 100 conidia/larvae (1 × 10^4^ conidia/mL) of *A. fumigatus* at the same site of injection. Preliminary experiments using 10^3^ and 10^6^ conidia/larvae showed the early death of the larvae (2 days) after conidia inoculum. With the above-mentioned concentration of 100 conidia/larvae, the larvae were able to survive longer, and we could perform the survival analysis clearly. The larvae were kept at 37 °C in the dark. Mortality was monitored daily; a lack of movement after physical stimuli was indicative of larva death. We performed a test to verify that neither the inoculum volume nor the site of injection was the cause of larva death. The results showed that despite the inoculum volume (57 µL) and the site of the second injection (the same proleg) there was no increase in larvae mortality.

### 2.14. Statistical Analysis

The results are expressed as the mean ± SEM of the three independent experiments. The statistical analysis was carried out using the Graph Pad Prism software version 8.4.3 (GraphPad Software, San Diego, CA, USA). Homogeneous variance was analyzed, and the difference between the means of the groups was calculated by analysis of variance (one-way) and Dunnett’s test thereafter. In addition, a two-tailed unpaired *t* test was used for the parametric data. For comparisons of survival curves, log rank (Mantel–Cox) tests were used. Differences with *p* < 0.05 were considered statistically significant.

## 3. Results

### 3.1. A. fumigatus EVs Induce TNF-α by Macrophages

**Figure 1:**
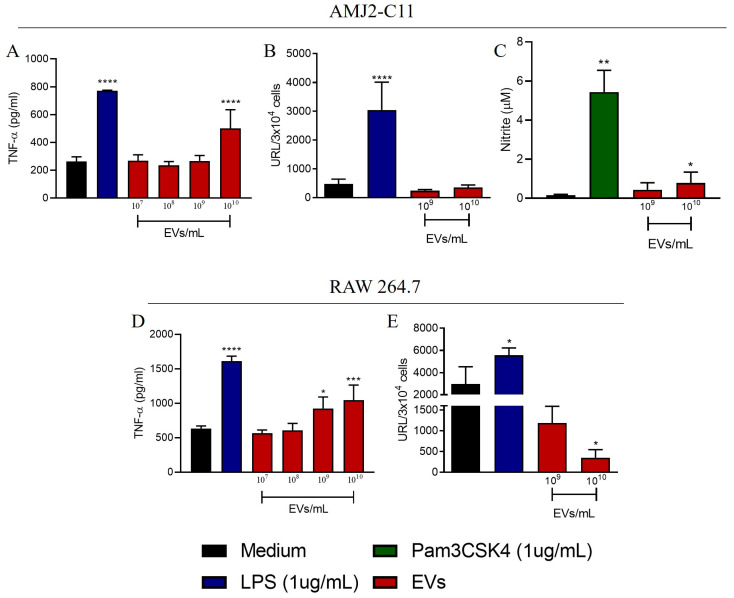
Production of inflammatory mediators by macrophages stimulated with EVs. AMJ2-C11 and RAW 264.7 macrophages were stimulated with EVs (10^7^ to 10^10^ EVs/mL) for 24 h. For AMJ2-C11 macrophages, TNF-α (**A**), O_2_^•−^ (**B**), and NO (**C**) were measured in the supernatant. For RAW 264.7 macrophages, TNF-α (**D**) and O_2_^•−^ (**E**) were measured. The results are expressed as mean ± SEM and were compared to medium through one-way analysis of variance followed by Dunnett’s test. **** *p* < 0.0001, *** *p* < 0.001, ** *p* < 0.01 and * *p* < 0.05.

Fungal EVs transport several molecules that up- or down-modulate inflammation and immune responses. To clarify the *in vitro* inflammatory properties of *A. fumigatus* EVs, we analyzed the AMJ2-C11 and RAW 264.7 macrophage lines stimulated with different concentrations of *A. fumigatus* EVs (10^7^ to 10^10^ EVs/mL) for 24 h. In parallel, those cells were stimulated with LPS, as a positive control, or were left unstimulated (medium). AMJ2-C11 macrophages showed significant and increased production of TNF-α at the highest concentration of EVs (10^10^ EVs/mL) compared to the unstimulated cells (medium), which were not statistically different from the LPS-stimulated cells (Figure 1A). The production of IL-6 was not altered (Figure S1A). Similar results were observed with RAW 264.7 cells; TNF-α production was increased upon stimulation with 10^9^ and 10^10^ EVs/mL (Figure 1D), but no alterations in IL-6 and IL-1β production were observed (Figure S1B,C).

Nitric oxide (NO) and superoxide (O_2_^•−^) play important roles in killing invading organisms, acting in phagolysosomes inside phagocytes. O_2_^•−^ is involved in host defense against *A. fumigatus* infection *in vivo* [26]. After 24 h of stimulation with *A. fumigatus* EVs at the highest concentration (10^10^ EVs/mL), AMJ2-C11 macrophages exhibited increased NO production (Figure 1C) and no O_2_^•−^ production (Figure 1B). RAW 264.7 macrophages showed reduced O_2_^•−^ production after stimulation with 10^10^ EVs/mL (Figure 1E).

### 3.2. A. fumigatus EVs Induce Lower Arginase-1 and Higher iNOS Transcription in RAW 264.7 Macrophages

**Figure 2:**
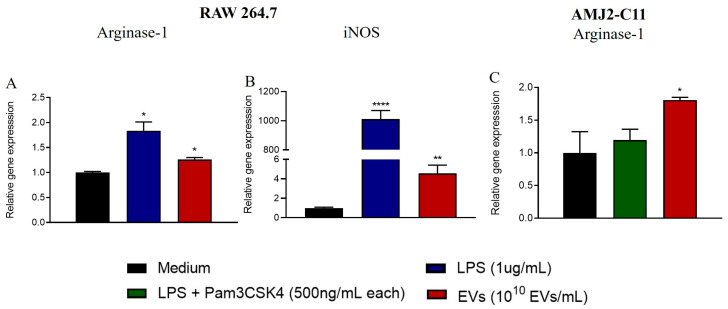
RAW 264.7 macrophages, but not AMJ2-C11, display polarization toward an M1 profile after stimulation with EVs. After 24 h of stimulation with 10^10^ EVs/mL, mRNA was extracted from AMJ2-C11 and RAW 264.7 macrophages. qPCR was performed to assess the expression of polarization signature genes. Panels A and B show the relative expression of arginase-1 (**A**) and iNOS (**B**) of RAW 264.7 macrophages. Panel (**C**) shows the relative expression of arginase-1 in AMJ2-C11 macrophages after 24 h of treatment. The results are expressed as mean ± SEM and were compared to the medium through an unpaired two-tailed *t* test. T test. **** *p* < 0.0001, ** *p* < 0.01 and * *p* < 0.05.

Activated macrophages or M1 macrophages, which express the iNOS enzyme and produce proinflammatory mediators, are effective for killing *A. fumigatus* [27,28]; anti-inflammatory or M2 macrophages that express arginase 1 (Arg1) also have a phagocytic profile in an *in vivo* infection, but their killing capabilities have not been addressed [29]. To address the polarization status of macrophages in response to *A. fumigatus* EVs, we analyzed their transcriptional gene profile. AMJ2C-11 and RAW 264.7 cells were stimulated for 24 h with 10^10^ *A. fumigatus* EVs/mL. Both mRNA levels of iNOS and arginase-1 were increased in RAW 264.7 cells after incubation with *A. fumigatus* EVs (Figure 2A,B). Only arginase-1 had increased gene expression in AMJ2-C11 cells (Figure 2C).

### 3.3. A. fumigatus EVs Augment Adhesion Molecule Gene Expression in RAW 264.7 Macrophages

**Figure 3:**
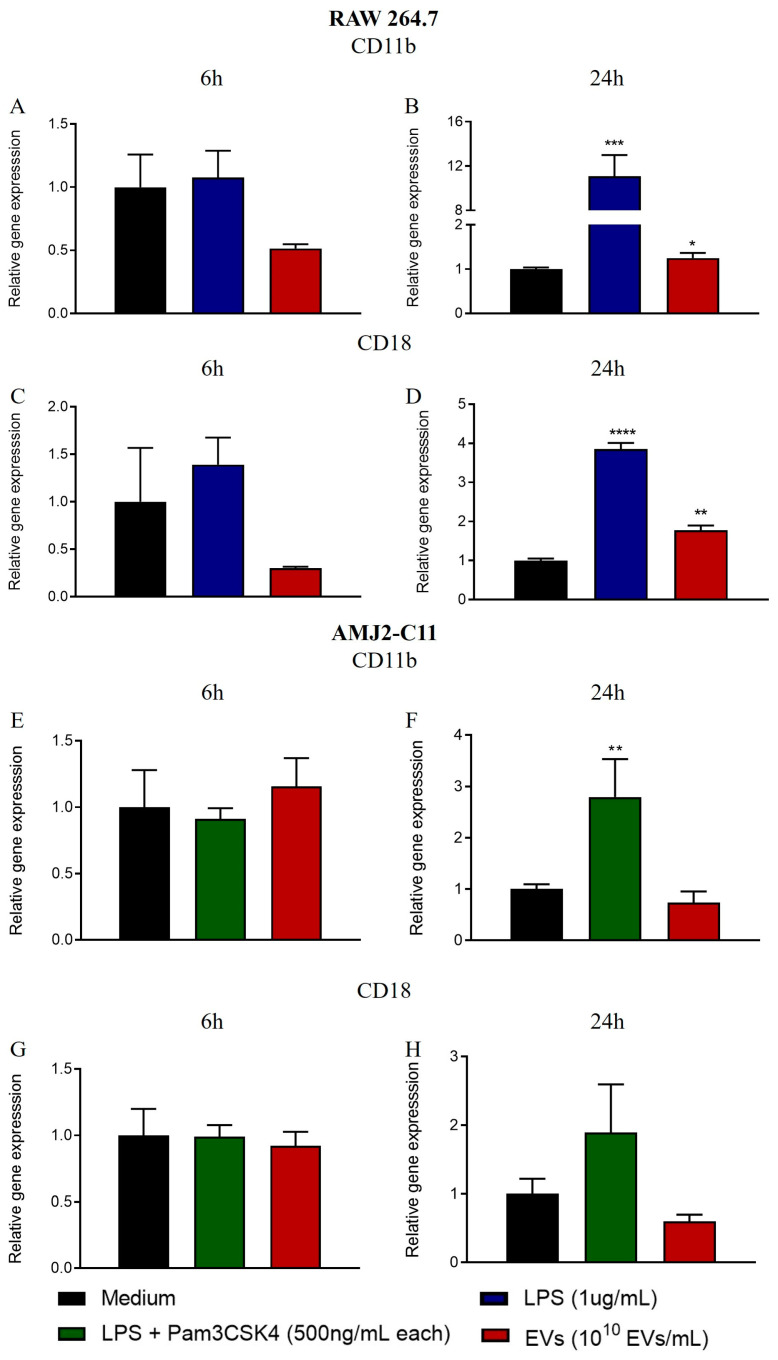
RAW 264.7 macrophages, but not AMJ2-C11, show increased mRNA levels in adhesion molecules after stimulation with EVs. AMJ2-C11 and RAW 264.7 cells were treated with 10^10^ EVs/mL for 6 and 24 h. The relative expression of CD11b and CD18 was assessed by qPCR. The relative expression of CD11b and CD18 after 6 and 24h of treatment in RAW 264.7 cells is shown in panels (**A**–**D**). The relative expression of CD11b and CD18 after 6 and 24 h of treatment in AMJ2-C11 cells is shown in panels (**E**–**H**). The results are expressed as mean ± SEM and were compared to the medium through an unpaired two-tailed *t* test. T test. **** *p* < 0.0001, *** *p* < 0.001, ** *p* < 0.01, and * *p* < 0.05.

Optical microscopy revealed the altered morphology of RAW 264.7 cells stimulated with *A. fumigatus* EVs for 24 h (Figure S2). The cells displayed more dendritic-like protrusions and adhered more avidly to the flask bottom, suggesting increased production of adhesion molecules. Treatment with EVs for 6 h had no influence on CD11b or CD18 transcript levels (Figure 3A,C), while increased CD11b and CD18 expression levels were detected after 24 h stimulation (Figure 3B,D). CD11b and CD18 form the alpha and beta chain of the Macrophage-1 antigen (Mac-1), respectively, which mediates cellular adhesion and other responses [30]. The optical microscopy results were consistent with qPCR results. Regarding AMJ2-C11 cells stimulated with *A. fumigatus*, EVs did not display morphological changes, and CD11b and CD18 transcript levels were not altered at any time point (Figure 3E–H). Using an adhesion assay with plates coated with fibrinogen, the ligand for Mac-1 [30], we showed that RAW 264.7 macrophages stimulated with *A. fumigatus* EVs showed no difference between treatment with EVs and unstimulated cells (medium); both showed similar percentages of reduced Resazurin (Figure S3). LPS induced increased adhesion of RAW 264.7 cells, as demonstrated by the higher percentage of reduced Resazurin, which correlated with the attachment of more cells in the wells (Figure S3).

### 3.4. A. fumigatus EVs Fail to Induce NETs Release and Cytokine Production by Neutrophils

**Figure 4:**
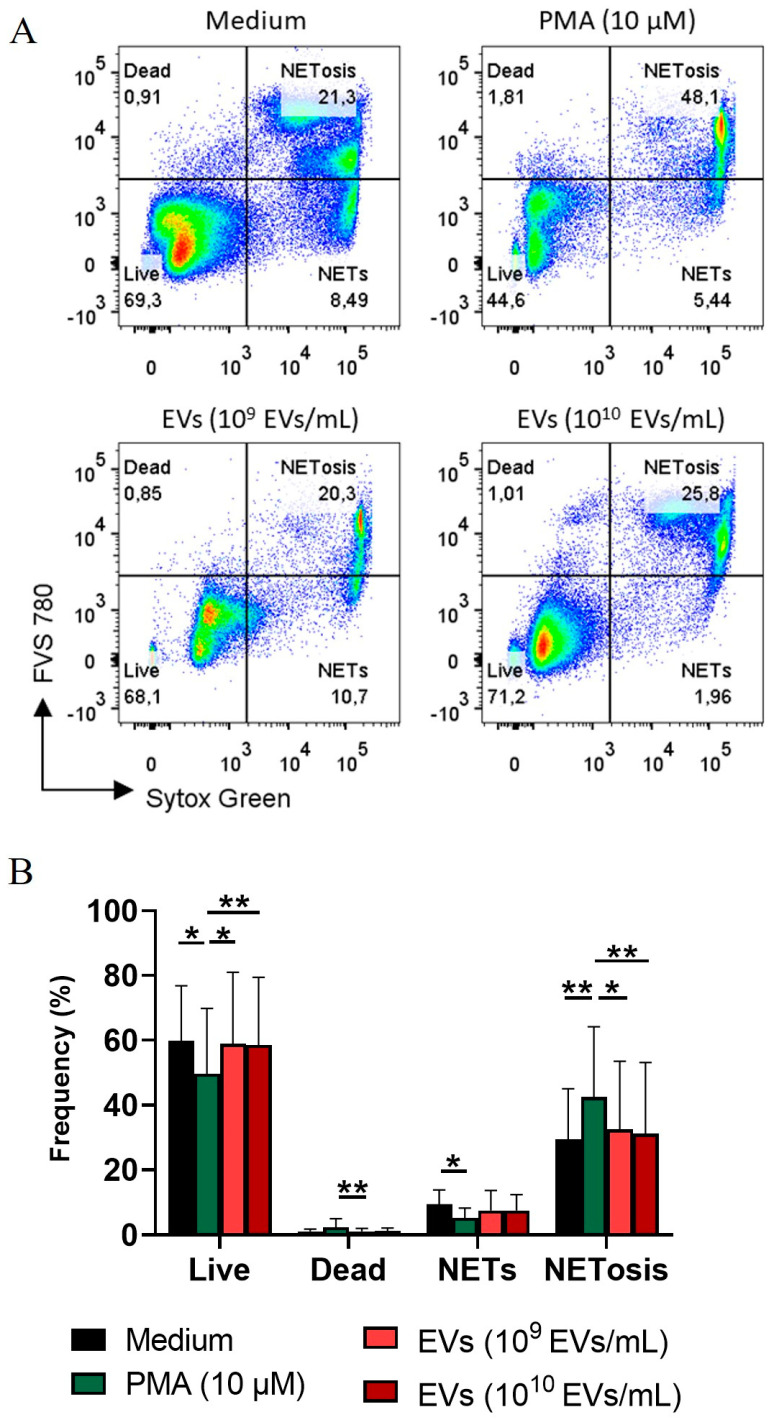
EVs do not induce NETosis in human neutrophils. Human neutrophils were stimulated with EVs or phorbol myristate acetate (PMA). Representative dot plot of neutrophils stimulated with PMA (positive control) or 10^9^ or 10^10^ EVs/mL (**A**). Percentage of live cells (FVS780−Sytox−), dead cells (FVS780+Sytox−), NETs (FVS780−Sytox+), and NETosis (FVS780+Sytox+) (**B**). Data are representative of three experiments (*n* = 9/group) and are expressed as mean ± SD. * *p* < 0.05; ** *p* < 0.001.

**Figure 5:**
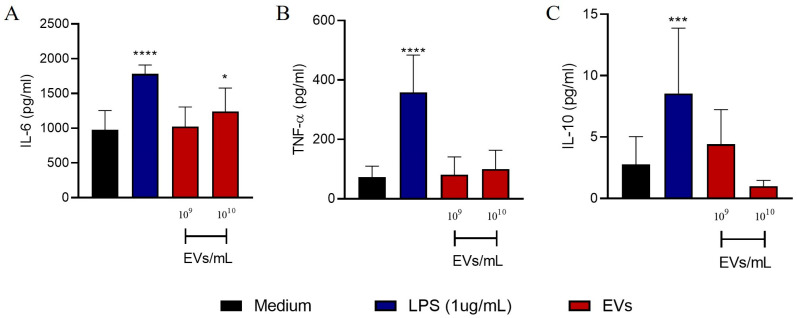
EVs induce the release of IL-6 by human PBMCs. PBMCs (2 × 10^5^ cells/well) were stimulated with 10^9^ or 10^10^ EVs/mL for 24 h. The production of IL-6 (**A**), TNF-α (**B**), and IL-10 (**C**) was measured in the supernatant. The results are expressed as mean ± SEM and were compared to the medium through one-way analysis of variance followed by Dunnett’s test. **** *p* < 0.0001, *** *p* < 0.001, and * *p* < 0.05.

To evaluate the effect of *A. fumigatus* EVs on the release of neutrophil extracellular traps (NETs), neutrophils isolated from the blood of healthy donors were stimulated with *A. fumigatus* EVs. Flow cytometry analysis confirmed that stimulation with (PMA) as the positive control induced the release of NETs and promoted neutrophil death, a mechanism known as NETosis. Increased NETosis or the release of NETs was not evident when neutrophils were stimulated with 10^9^ or 10^10^ *A. fumigatus* EVs/mL was compared with the negative control (Figure 4A,B). Moreover, *A. fumigatus* EVs did not induce IL-6 and IL-1β production by human neutrophils (Supplementary Material). The results suggest that *A. fumigatus* EVs neither induce NET release nor alter cytokine production by human neutrophils. We also assessed the production of IL-6, TNF-α, and IL-10 by human PBMCs treated with *A. fumigatus* EVs for 24 h. IL-6 secretion was increased due to 10^10^ EVs/mL (Figure 5A); however, EVs did not induce TNF-α or IL-10 production (Figure 5B,C).

### 3.5. EVs Induce Increased Survival in Galleria Mellonella Model of A. fumigatus Infection

**Figure 6:**
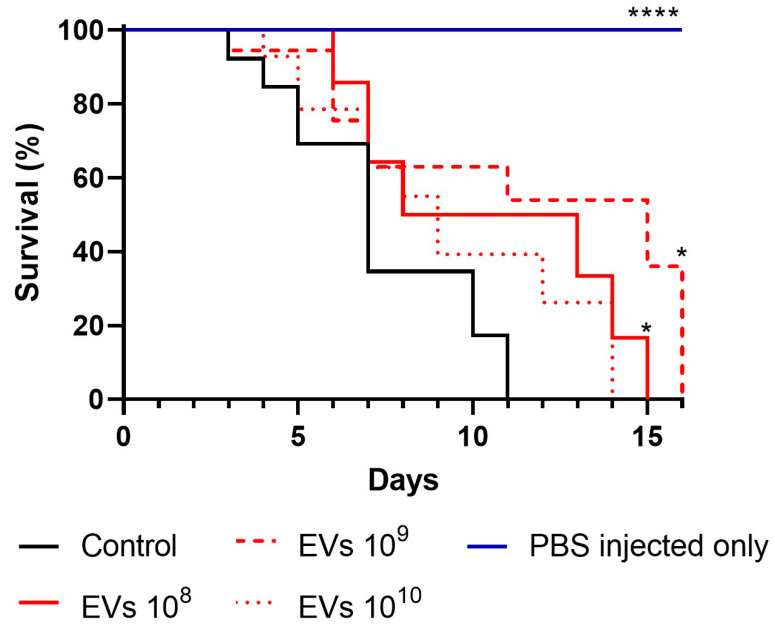
Treatment of *G. mellonella* with EVs increases larvae survival after fungal challenge. *G. mellonella* larvae were treated with 10^8^, 10^9^, or 10^10^ EVs. After 48 h, each larva was infected with 100 conidia of *A. fumigatus*. Larva survival was assessed daily until all larvae were dead. Log rank (Mantel–Cox) test for survival curve analysis. **** *p* < 0.0001, * *p* < 0.05. The PBS-injected-only group were injected with 57 µL of PBS in the last left proleg; after 48 h, 10 µL of PBS was injected at the same site, and mortality was recorded.

Since *A. fumigatus* EVs were unable to induce a robust proinflammatory response from innate leukocytes and human PBMCs, we assessed whether *A. fumigatus* EVs could influence *G. mellonella* survival in an *in vivo* model of infection. *G. mellonella* has an immune system that, in some respects, shows similarities to the mammal innate immune system which can be used to explore the interaction of fungal pathogens and the host [31]. *G. mellonella* larvae were treated with 10^8^ to 10^10^ *A. fumigatus* EVs/larvae for 48 h and were then infected with *A. fumigatus* conidia. In the control group (PBS-treated), all larvae were dead by day 11. In contrast, survival was greater among the treated larvae (Figure 6). Treatment with 10^8^ or 10^9^ EVs significantly increased the survival of *G. mellonella*, leading to death only by days 15 and 16; this indicates the protective effect of *A. fumigatus* EVs *in vivo*.

## 4. Discussion

Several biomolecules transported by EVs are related to fungal survival, virulence, and immune evasion [32]. Herein we investigated the *in vitro* pro- and anti-inflammatory properties of *A. fumigatus* EVs. A previous study by our group showed that treatment with *A. flavus* EVs induced the production of TNF-α, IL-6, IL-1β, and NO by bone marrow-derived macrophages (BMDMs) [19]. *A. flavus* EV-treated BMDMs showed an increased phagocytic index and intracellular killing abilities, which were related to an M1 macrophage polarization [19].

In this study, we used AMJ2-C11 and RAW 264.7 macrophages. AMJ2-C11 is an alveolar macrophage line. Thus, AMJ2-C11 cells would mimic the first phagocytes to interact with EVs *in vivo*, while RAW 264.7 macrophages would mimic the contact of EVs in a disseminated infection. Our results show a lower pro-inflammatory profile for *A. fumigatus* EVs compared to *A. flavus* EVs. Both macrophage cell lines displayed increased TNF-α production only when stimulated with the highest concentrations of EVs (10^9^ and 10^10^ EVs/mL). NO production was only increased in AMJ2-C11 macrophages stimulated with the highest concentration of EVs (10^10^ EVs/mL). Superoxide was not induced by EVs in AMJ2-C11 cells, and a decrease was noted in RAW 264.7 cells, even when stimulated with the highest concentration of EVs compared to the control. The results suggest that *A. fumigatus* EVs may act differentially in both macrophage lines tested. In AMJ2-C11 alveolar macrophages derived from C57BL6J mice, EVs induced an increased expression of arginase-1 mRNA with no increased expression of iNOS mRNA. RAW 264.7 macrophages derived from the ascites of BALB/c mice displayed increased expression of arginase-1 and iNOS mRNA. Souza et al., 2019 also studied *A. fumigatus* EVs. They used the *A. fumigatus* A1163 strain grown in a YG liquid medium (yeast extract powder, glucose, and trace elements), while we have used the *A. fumigatus* CEA17 strain grown in a solid minimal medium. The nutrition status of the medium influences the fungal EVs’ profile. Fungal species can produce different EVs with altered size and cargo depending on growth media [33]. EVs from *Histoplasma capsulatum* cultured in a rich medium (supplemented Ham’s F-12) have double protein content, 18 times more sterol content, and altered protein expression when compared to a less nutritional medium such as RPMI-1640 [33]. Despite the differences in growth conditions and strain, Souza et al., 2019 showed increases in both the phagocytosis and fungal killing activities of RAW 264.7 macrophages, as well the production of TNF-α and chemokine CCL2, but not IFN-γ or IL-10 [34]. A similar response was noted when bone marrow-derived neutrophils (BMDNs) were tested, increasing phagocytosis and fungal killing but not prompting the production of TNF-α, IL-1β or IFN-γ [34]. Thus, these findings suggest that albeit the difference in the strain and culture conditions, EVs from *A. fumigatus* promote low pro-inflammatory cytokine production *in vitro* by some innate leukocytes. The production of inflammatory mediators after the interaction of *A. fumigatus* conidia with alveolar macrophages is necessary to convey the message to leukocytes indicating a microbe invasion. TNF-α secreted by alveolar macrophages is critical for fungal clearance [35], while NO and superoxide production within phagolysosomes are essential for fungal killing [26,27]. Secreted EVs may act as an immunomodulatory component of the invading *A. fumigatus* conidia. In this sense, it was demonstrated that immunization with *A. fumigatus* EVs could partially protect mice from fungal infection by evoking a less pronounced inflammation [36]. After the *A. fumigatus* conidia challenge, mice immunized with EVs displayed an immune regulatory profile in the lungs, evident as a reduced influx of inflammatory cells and primarily neutrophils and reduced vascular permeability. Furthermore, immunized animals produced fewer inflammatory cytokines in the lungs 48 h after infection, with increasing conidia phagocytosis and killing by neutrophils compared to non-immunized mice [36]. However, despite the less inflammatory profile of the EV-immunized mice, no difference in survival was observed when compared to the non-immunized ones [36]. Differing from this study, the above-mentioned study was performed using EVs from *A. fumigatus* strain A1163 cultivated in a liquid YG medium. Similar to the macrophage response, EVs failed to induce the production of IL-6 or IL1-β by human neutrophils. The production of TNF-α and IL-1β by BMDNs was reportedly achieved only when the cells were treated with EVs and infected by *A. fumigatus in vitro* [34]. The findings demonstrate a priming role for *A. fumigatus* EVs. Likewise, when human PBMCs were treated with EVs, only IL-6 production was induced at the highest concentration of EVs, but no increased production of TNF-α or IL-10 was noted.

Fungal EVs can modulate innate cells [15]. *Candida albicans* EVs can induce the production of inflammatory mediators such as IL-12p40, TNF-α, IL-6, and NO by BMDCs, BMDMs, and RAW 264.7 cells [37,38]. *C. auris* EVs increased the production of IL-6 by BMDC [39]. *C. neoformans* EVs increased the production of TNF-α, TGF-β, IL-6, and NO by RAW 264.7 macrophages [40]. *P. brasiliensis* EVs induce a strong proinflammatory response by murine peritoneal macrophages with the production of IL-12p40, IL-12p70, IL-6, TNF-α, IL-1α, IL-1β, and NO, which was related to an M1 macrophage polarization [18]. Similarly, *T. interdigitale* EVs can induce inflammatory mediators by BMDMs, which include TNF-α, IL-6, IL-1β, and NO, also displaying M1 macrophage polarization. *H. capsulatum* EVs impair the phagocytosis and fungal killing of stimulated BMDMs [20]. Thus, fungal EVs play different roles in activating or modulating innate responses. Our group has summarized these properties elsewhere [15].

RAW 264.7, but not AMJ2-C11 cells, stimulated with *A. fumigatus* EVs displayed an altered morphology with the formation of elongated protrusions, suggesting augmented expression of adhesion molecules. Indeed, we demonstrated increased mRNA levels of CD11b and CD18 integrins in EV-stimulated RAW 264.7 cells. In contrast, an adhesion assay showed no difference comparing the negative control to EV-treated cells. Due to the nature of the assay, the sensitivity may be not high enough to capture subtle changes in the expression of adhesion molecules. Therefore, further studies are necessary to definitively understand whether *A. fumigatus* EVs influence the expression and function of adhesion molecules.

Integrins are involved in several steps in phagocyte function. They modulate adhesion to extracellular matrix, phagocytosis, migration, and spreading [41]. An *in vitro* assay demonstrated that peritoneal polymorphonuclear neutrophils (PMNs) use Mac-1 or CD11b (*α*_M_*β*_2_), while peritoneal activated macrophages use Mac-1 and complement receptor 4 (CR4) (*α*_X_*β*_2_) to engulf and kill germinated *C. albicans* cells [42]. *In vivo* experiments with *C. albicans* infection demonstrated that Mac-1 is required for the elimination of invading fungi by PMNs, while CR4 is necessary for macrophage fungal killing [42]. Additionally, Mac-1 is important for the adhesion and internalization of *A. fumigatus* conidia into alveolar epithelial cells [43] and plays a pivotal role in NET formation by human neutrophils [44,45].

Conidia from *A. fumigatus* induce NET formation by human neutrophils [44,45]. In the present study, EVs failed to induce NET formation by human neutrophils. NETs released by neutrophils do not directly kill *A. fumigatus* [45]. However, NETs are a powerful tool to promote the control of the hyphal form of *A. fumigatus* by virtue of their fungistatic activity [46,47].

*G. mellonella* is an established invertebrate model of fungal infection, as its immune system shares some similarities with mammal innate immunity [31,48]. The *G. mellonella* hemolymph, which is analogous to human blood, contains hemocytes. Hemocytes are immune cells capable of many functions, including phagocytosis, nodulation, and encapsulation [49]. Accordingly, *G. mellonella* larvae are used to study many fungal infections [50,51,52,53,54,55]. Here we demonstrate that treatment with EVs prior to the *A. fumigatus* conidia infection of *G. mellonella* protected the larvae from early death. *G. mellonella* lack adaptive immunity [31]. Thus, the observed outcome must reflect the activation of hemocytes, which in turn partially protect larvae. As discussed above, Souza et al., 2022 demonstrated the EVs’ vaccinal properties from *A. fumigatus* in a pulmonary aspergillosis mice model, probably by engaging the adaptive arm of immunity. Our work shows that the activation of *G. mellonella* immune cells by *A. fumigatus* EVs resulted in increased larva survival, suggesting that *A. fumigatus* EVs could promote an immune cell activation that protects the larvae. Other fungal EVs have been used in a similar manner to study the protection conferred by these vesicles. Our group showed that treatment with *A. flavus* EVs resulted in a reduced CFU count and increased survival of *G. mellonella* larvae after *A. flavus* infection [19]. We observed a similar survival curve in PBS-treated larvae after *A. fumigatus* or a *A. flavus* infection, with larvae infected with *A. fumigatus* conidia showing slightly earlier death. Previous reports showed the opposite: in one case, *A. flavus* showed an increased pathogenicity in *G. mellonella* larvae when compared to *A. fumigatus* or *A. nidulans* [56]. Although the same number of *A. flavus* or *A. fumigatus* conidia per inoculum was used in both studies (100 conidia per larvae), in this study a much higher concentration of EVs was capable of conferring protection for the larvae after fungal infection. *C. albicans* EVs did not affect larva survival but significantly decreased the fungal burden of *G. mellonella*-infected larvae [37]. Interestingly, *G. mellonella* treatment with *C. neoformans* EVs resulted in the early death of treated larvae [57].

In summary, this work shows that *A. fumigatus* EVs induce a partial pro-inflammatory response by mouse macrophage lines, human neutrophils, and PBMCs. Also, *A. fumigatus* EVs act differently in macrophage lines, probably because those macrophages express a differential density of pattern recognition receptors. Finally, EVs protect *G. mellonella* larvae in an infection model, demonstrating its effects over innate cells responsible for the larvae protection. This work contributes to elucidating the inflammatory properties of EVs, which may account for the modulation of immune responses and possibly the control of infection. The understanding of initial steps in the crosstalk between *A. fumigatus* EVs and innate leukocytes might drive the identification of critical targets for the development of new anti-fungal therapies and adjuvants.

## Figures and Tables

**Table 1:** Primers used in qPCR.

Gene	Sequence 5′–3′	Concentration (nM)	Efficiency (%)	Ref.
iNOS2	FWD: CCGAAGCAAACATCACATTCA REV: GGTCTAAAGGCTCCGGGCT	70	110.11	[18]
arginase-1	FWD: GTTCCCAGATGTACCAGGATTC REV: CGATGTCTTTGGCAGATATGC	100	99.33	[18]
CD11b	FWD: TACTTCGGGCAGTCTCTGAGTG REV: ATGGTTGCCTCCAGTCTCAGCA	300	94	[22]
CD18	FWD: CTTTCCGAGAGCAACATCCAGC REV: GTTGCTGGAGTCGTCAGACAGT	300	105.5	[23]
Gapdh	FWD: GGTGCTGAGTATGTCGTGGA REV: CGGAGATGATGACCCTTTTG	300	108.76	[24]

The term efficiency (%) is related to the efficiency of the qPCR reaction, considering the kinetics of the reaction, assuming a twofold increase in amplicon in each cycle for 100% efficiency. In general, PCR efficiency should be 90–110% for target and normalizing genes, which ensures accurate quantification. To verify this information, a standard curve was performed using two-fold serial dilutions; the reaction efficiency for each pair of primers is provided within the table.

## Data Availability

Not applicable.

## Supplementary Materials

The following supporting information can be downloaded at: https://www.mdpi.com/article/10.3390/jof9050541/s1, Figure S1: EVs fail to induce the production of IL-6 and IL-1β by macrophages.; Figure S2: EVs induce membrane extended processes in RAW 264.7 macrophages.; Figure S3: EVs fail to increase macrophage adhesion.
