# Supplementary material for: Aspergillus fumigatus Extracellular Vesicles Display Increased Galleria mellonella Survival but Partial Pro-Inflammatory Response by Macrophages
Source: J Fungi (Basel). 2023 May 4;9(5):541. doi: 10.3390/jof9050541 (PMC10218922; doi:10.3390/jof9050541)
Supplement: Supplementary file 1 [file jof-09-00541-s001.zip › jof-2235137-supplementary.pdf]

Supplementary Material

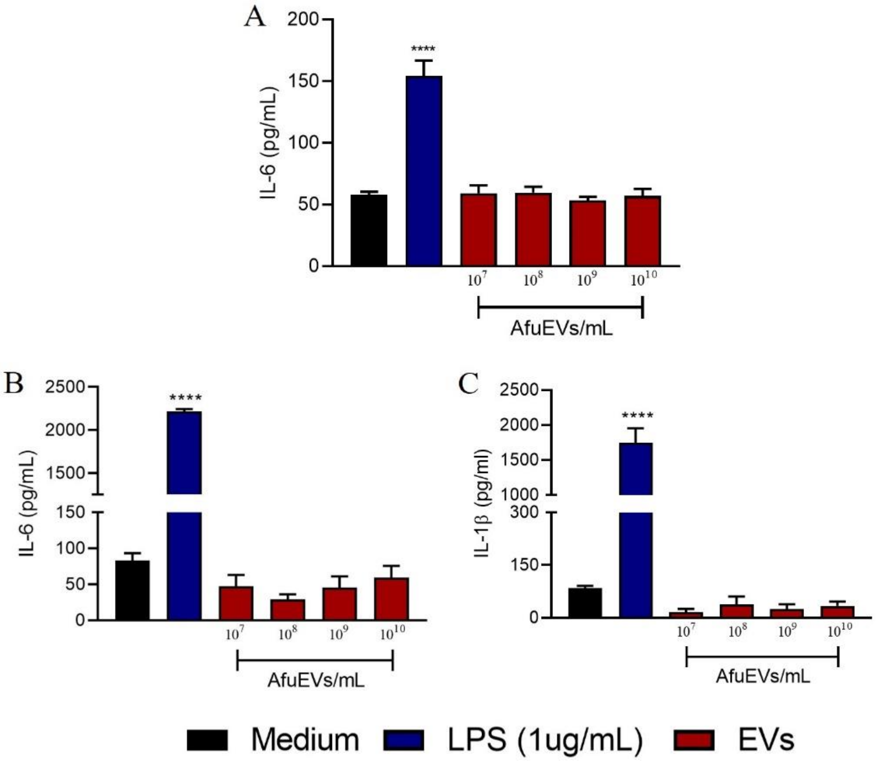

Figure S1. EVs fail to induce the production of IL-6 and IL-1β by macrophages. AMJ2-C11 (A) and RAW 264.7 (B and C) macrophages were stimulated with EVs (107 to 1010 EVs/mL) for 24 h. The presence of IL-6 and IL-1β in the supernatant was determined. The results are expressed as mean SEM, and were compared to medium through one-way analysis of variance followed by Dunnett's test. **** $p < 0.0001$.

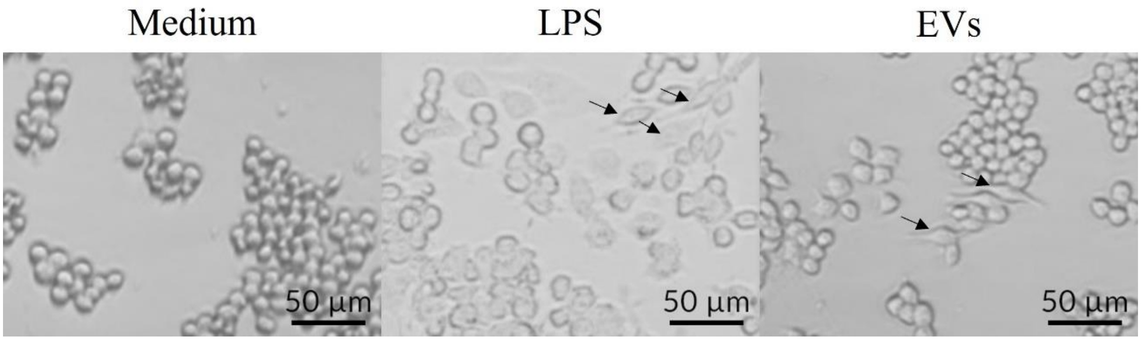

Figure S2. EVs induce membrane extended processes in RAW264.7 macrophages. RAW264.7 cells were stimulated for 24 h with medium alone, 1010 EVs/mL, or LPS (1 µg/mL). Cells stimulated with EVs developed membrane protrusions indicating cellular activation and augmented expression of adhesion molecules. Black arrows show cells with membrane protrusions.

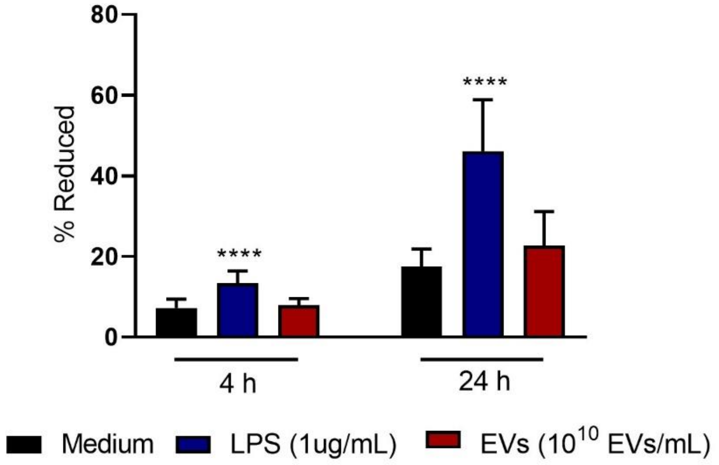

Figure S3. EVs fail to increase macrophage adhesion. RAW 264.7 macrophages were cultured in fibrinogen coated plates and stimulated with LPS (1 μg/mL) or 1010 EVs/mL. After washing with PBS, the remaining cells were incubated with Resazurin for detection of attached cells. The results are expressed as mean SEM, and were compared to medium through one-way analysis of variance followed by Dunnett's test. **** $p < 0.0001$.
